# Inflammatory Response to Regulated Cell Death in Gout and Its Functional Implications

**DOI:** 10.3389/fimmu.2022.888306

**Published:** 2022-04-06

**Authors:** Jianan Zhao, Kai Wei, Ping Jiang, Cen Chang, Lingxia Xu, Linshuai Xu, Yiming Shi, Shicheng Guo, Yu Xue, Dongyi He

**Affiliations:** ^1^ Guanghua Clinical Medical College, Shanghai University of Traditional Chinese Medicine, Shanghai, China; ^2^ Department of Rheumatology, Shanghai Guanghua Hospital, Shanghai University of Traditional Chinese Medicine, Shanghai, China; ^3^ Institute of Arthritis Research in Integrative Medicine, Shanghai Academy of Traditional Chinese Medicine, Shanghai, China; ^4^ Computation and Informatics in Biology and Medicine, University of Wisconsin-Madison, Madison, WI, United States; ^5^ Department of Medical Genetics, School of Medicine and Public Health, University of Wisconsin-Madison, Madison, WI, United States; ^6^ Department of Rheumatology, Huashan Hospital, Institute of Rheumatology, Immunology and Allergy, Fudan University, Shanghai, China; ^7^ Arthritis Institute of Integrated Traditional and Western Medicine, Shanghai Chinese Medicine Research Institute, Shanghai, China

**Keywords:** gout, inflammation, programmed cell death, pyroptosis, NETosis, necroptosis, apoptosis

## Abstract

Gout, a chronic inflammatory arthritis disease, is characterized by hyperuricemia and caused by interactions between genetic, epigenetic, and metabolic factors. Acute gout symptoms are triggered by the inflammatory response to monosodium urate crystals, which is mediated by the innate immune system and immune cells (e.g., macrophages and neutrophils), the NACHT, LRR, and PYD domains-containing protein 3 (NLRP3) inflammasome activation, and pro-inflammatory cytokine (e.g., IL-1β) release. Recent studies have indicated that the multiple programmed cell death pathways involved in the inflammatory response include pyroptosis, NETosis, necroptosis, and apoptosis, which initiate inflammatory reactions. In this review, we explore the correlation and interactions among these factors and their roles in the pathogenesis of gout to provide future research directions and possibilities for identifying potential novel therapeutic targets and enhancing our understanding of gout pathogenesis.

## Introduction

Gout is a type of arthritis associated with inflammation and is primarily caused by the deposition of monosodium urate (MSU) crystals in joints because of increased serum uric acid levels. Elevated serum uric acid levels gradually develop into hyperuricemia and lead to MSU formation, which is stimulated by various factors and leads to joint inflammation. The risk factors for gout include genetic, metabolic, and multiple comorbidities, including metabolic syndrome, cardiovascular disease, and renal disease (1). The global healthcare burden of gout has increased. Worldwide prevalence and incidence vary by study region, population, and assessment methods, ranging from <1% to 6.8%, with an incidence of 0.58–2.89 per 1,000 person-years (1). Patients with gout are primarily treated with uric acid-lowering and anti-inflammatory therapies (1, 2). Uric acid-lowering drugs such as allopurinol, febuxostat, probenecid sulfinpyrazone, and benzbromarone—typically used in combination—increase the metabolic burden on the kidney, the risk of kidney stone disease, and uric acid excretion and primarily reduce purine catabolism (3). Additionally, some patients do not accept long-term uric acid-lowering therapies due to lack compliance and, therefore, exhibit a poor treatment response. Anti-inflammatory treatments include nonsteroidal anti-inflammatory agents, colchicine, and corticosteroids. However, these drugs may induce multiple side effects; the development of new drugs has been slow (3). Additionally, currently used clinical drugs do not cover all clinical stages of gout.

Cell death commonly occurs in various physiological and pathological processes in the human body. In 2018, the Nomenclature Committee on Cell Death classified cell death as either accidental or regulated based on functional differences, which includes 11 primary forms: apoptosis, necroptosis, pyroptosis, ferroptosis, parthanatos, entotic cell death, NETotic cell death, autophagy-dependent cell death, alkaliptosis, oxeiptosis, and lysosome-dependent cell death (4). Numerous studies have characterized these types of cell death and their underlying molecular mechanisms. However, the role of cell death in certain diseases remains unclear. Different cell death patterns may be associated with the different clinical stages of gout. MSUs in gout can function as pattern recognition receptors and thus act as danger signals to activate multiple immune cells and multiple cell death pathways, possibly through mutual crosstalk (5). For example, tumor necrosis factor (TNF)-α and interleukin (IL)-1β are important molecules or products of apoptosis, necroptosis, and pyroptosis. These molecules can be secreted by monocytes and promote inflammatory cell infiltration in patients with gout (6–9). In this review, we provide insights that may be useful for developing new drugs for gout and for determining the associated pathological mechanisms by describing the multiple forms of cell death, including pyroptosis, NETosis, necroptosis, and apoptosis, in gout and their roles.

## Pyroptosis

Pyroptosis is a form of pro-inflammatory cell death (10). The various key molecules involved in pyroptosis include the inflammasome [apoptosis-associated speck-like protein containing a C-terminal caspase activation and recruitment domain (ASC), NLRP3, and pro-caspase1], members of the gasdermin family, caspase1, and caspases4/5/11, which mediate the non-classical pathway (11). The activation of the NLRP3 inflammasome hydrolyzes IL-1β precursor (pro-IL-1β), IL-18 precursor (pro-IL-18) and gasdermin D *via* caspase-1, resulting in the maturation and release of IL-18 and IL-1β and N-terminal fragments of gasdermin D. It results in gasdermin-mediated pore formation and membrane rupture, the release of cellular contents to mediate inflammation (11). The activation of the NLRP3 inflammasome and release of IL-1β are thought to be important in the progression of hyperuricemia to gout. Both soluble uric acid and MSU in gout act as damage-associated molecular patterns (12). Soluble uric acid level and MSU accumulation depend on mitochondrial reactive oxygen species (ROS) production and the Toll-like receptor (TLR)/myeloid differentiation primary response (MyD)88-nuclear factor kappa B (NF-κB) signaling pathway to activate the NLRP3 inflammasome and pro-IL-1β transcription, respectively. When inflammasomes are activated, pro-IL-1β is cleaved by caspase1, which promotes maturation and pyroptosis to cause the release of biologically active IL-1β (12). In the following section, we focus on the potential link between pyroptosis, which is a potential therapeutic target, and gout.

### NLRP3 Inflammasome Activation and IL-1β Release Promote Inflammation in Gout

In gout, high uric acid levels typically lead to hyperuricemia, inflammation, and other complications. Uric acid precipitates into MSU and triggers the assembly of multiple cells with activated NLRP3 inflammasome and the maturation and release of IL-1β (13, 14). Numerous biological mediators contribute to this process *via* various mechanisms. Experimental mice lacking critical components of pyroptosis (NLRP3, ASC, or caspase1) showed decreased knee neutrophil infiltration in response to MSU stimulation. Macrophages of these mice were defective in the MSU-induced IL-1β release process compared to those in the control group (15). Moreover, for monocyte/macrophage subsets in gout patients, granulocyte-macrophage colony-stimulating factor, G protein receptors (GPR43 and P2Y14), and microbial populations assist in inducing the expression of key molecules of MSU-promoted pyroptosis through different mechanisms, such as inhibition of cyclic adenosine monophosphate synthesis, production of metabolites, or inhibition of histone deacetylases activity (16–18). Cold-inducible RNA-binding protein, an endogenous damage-associated molecular pattern, promotes MSU-stimulated neutrophil infiltration, a CXC-motif receptor 2-dependent process associated with the NLRP3/ASC/caspase1/IL-1β/MyD88 pathway (19, 20).

**Figure 1:**
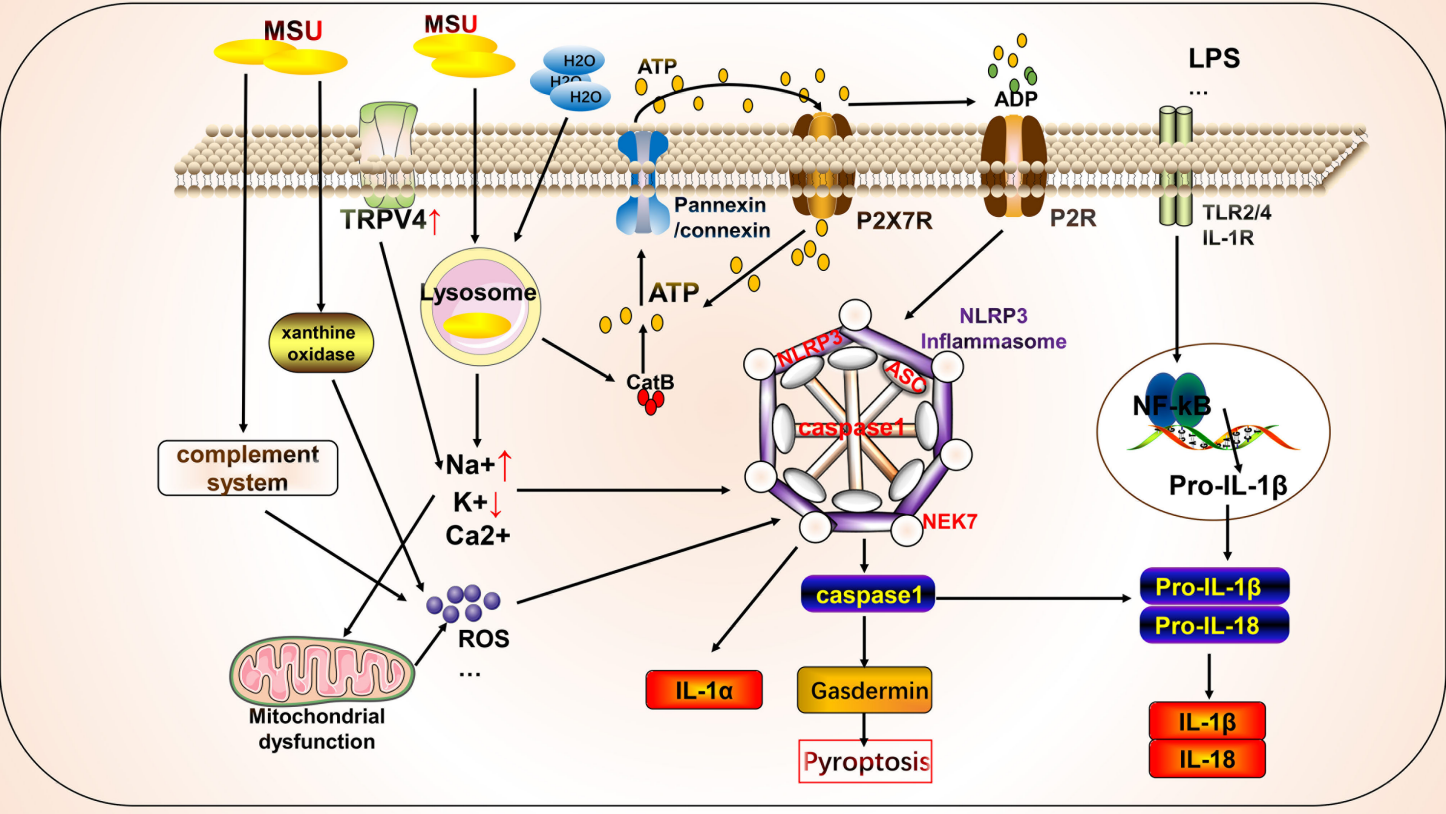
Mechanism of activation of key molecules for pyroptosis by monosodium urate (MSU) in gout. MSU can activate key molecules involved in pyroptosis, such as NLRP3 inflammasome, and promote the release of IL-1β through various mechanisms. MSU-stimulated destabilization of lysosomes leads to the release of cathepsin, which activates the NLRP3 inflammasome by regulating ATP metabolism through the cell surface pannexin/connexin channels and purinergic receptors. In addition, MSU can regulate intracellular ion concentrations and mitochondrial function, leading to the release and production of substances, such as ROS, to activate the NLRP3 inflammasome. The complement system, particularly the lectin system, is also partially involved in the activation of the NLRP3 inflammasome.

We further explored the mechanisms by which MSU activates the NLRP3 inflammasome, which can be described as follows: (**Figure 1**) (i) MSU is endocytosed by phagocytes and causes the release of histone B by disrupting lysosomes (21). It also releases intracellular ATP through the pannexin/connexin channels. Extracellular ATP is degraded by different extracellular ATPases to diphosphate and monophosphate and further degraded to adenosine or metabolites that interact with P2X/P2Y, which further increases ATP release and activates the NLRP3 inflammasome by activating purinergic receptors (P2X/P2Y) on the cell surface or in adjacent cells (22). Interestingly, histones B and S can directly cleave receptor interacting serine/threonine kinase 1 (RIPK1), a key molecule involved in necroptosis and apoptosis, thereby inhibiting macrophages from undergoing necroptosis or apoptosis and tending toward pyroptosis (23). In addition, Toll/IL-1 receptor-domain-containing adapter-inducing interferon-β and the apoptosis protein inhibitor IAP modulate the ubiquitination of receptor interacting serine/threonine kinase 3 (RIPK3) and mixed lineage kinase domain-like protein (MLKL) in lipopolysaccharide (LPS)-induced necroptosis *in vitro*. RIPK3 in IAP-deficient mice promotes autoantibody-mediated arthritis by promoting NLRP3 inflammasome formation and IL-1β release, which may be a consequence of RIPK3 inhibition of necroptosis by shifting the form of cell death to pyroptosis (24). The crosstalk between multiple cell death mechanisms requires further elucidation. (ii) MSU activates the NLRP3 inflammasome by regulating cell surface ion channel activity and intracellular and extracellular potassium ion concentrations upon contact with the macrophage surface independent of histone proteinase B (25). In addition, phagocytosis of MSU decreases intracellular K^+^ concentrations *via* a mechanism that may involve the low-pH environment of lysosomes, causing the release of abundant sodium, increased intracellular osmotic pressure, cell swelling, and decreased potassium ion concentration (26). We also found that the mechanosensitive transient receptor potential vanilloid 4 (TRPV4) channel expression level is upregulated in MSU-stimulated synovial macrophages and human peripheral blood mononuclear cells. Both genetic ablation and repression of TRPV4 attenuated MSU inflammation by inhibiting NLRP3 inflammasome and IL-1β production, suggesting that TRPV4 is required for MSU-mediated activation of the NLRP3 inflammasome cascade (27). TRPV4 is an osmotic cation channel. MSU-stimulated reduction in intracellular ATP levels leads to mitochondrial membrane depolarization, activating NLRP3 and causing IL-1β release through the regulation of K^+^- and Ca^2+^-mediated mitochondrial dysfunction (28). (iii) After MSU engulfment by the plasma membrane of phagocytes, MSU forms electrostatic bonds and lipid raft aggregations with cholesterol in the plasma membrane of dendritic cells, leading to further activation of the Syk/phosphatidylinositol-3-kinase (PI3K) signaling pathway by an intracellular immunoreceptor tyrosine-based activation motif (29). Activation of Syk signaling can lead to the production of ROS and promote the release of cytokines and chemokines that induce inflammation, possibly accompanied by NLRP3 activation (30, 31). Clec12a, a C-type lectin receptor widely expressed on the surface of innate immune cells, negatively regulates the Syk signaling pathway through an intracellular immunoreceptor tyrosine-based activation motif (32) and may inhibit MSU-induced cell death through a mechanism involving physical sensing of monosodium urate or recognition of a protein ligand of dead cells (33). (iv) The complement system also contributes to NLRP3 inflammasome activation. MSU can activate the complement component C5a to enhance ROS production, triggering NLRP3 inflammasome assembly and IL-1β release (34, 35). In response to C5a, neutrophils infiltrating the peritoneum release phosphatidylserine-positive neutrophil-derived microvesicles, thereby inhibiting the C5a response (34). Microvesicles also inhibit inflammation by releasing transforming growth factor-β through a phosphatidylserine/MerTK receptor-independent pathway (34). Intracellular C5a and C5a receptor 2 (C5aR2) interactions amplify double-stranded DNA-dependent protein kinase R expression *via* the mitogen-activated protein kinase/extracellular signal-regulated kinase pathway and type I interferon signaling. A deficiency in C5aR2 inhibits NLRP3 inflammasome activation and high-mobility group box 1 release from mouse macrophages (36). The function of C5aR2 varies among cells and can negatively regulate C5aR1 expression in T cells (37). Intracellular C5 and C5a receptor 1 (C5aR1) stimulate NLRP3 assembly and initiate caspase1-dependent IL-1β secretion, IFN-γ production, and Th1 differentiation in human CD4^+^ T cells (37). C-reactive protein was found to be the second most abundant protein on the surface of the MSU crystals. Both erythrocyte sedimentation rate and C-reactive protein levels are significantly elevated in elderly patients with gout during acute attacks (38). C-reactive protein recruits complement component C1 and mannose-binding lectin pathway protease MASP1 on the surface of MSU and enhances binding of C3 and terminal complement complexes, suggesting that the activation of the complement cascade pathway contributes to the subsequent cell death pathway; however, further studies are needed to verify this hypothesis (39). (v). MSU promoted NLRP3 inflammasome activation and IL-1β release by affecting mitochondrial function and oxidative stress. Mitochondria are important organelles that maintain intracellular homeostasis (40). Analysis of the effect of mitochondrial genetic variation and copy number variants on gout susceptibility showed that a reduced mtDNA copy number significantly increased the risk of gout. Mitochondria are important for the colocalization of NLRP3 and ASC and release of IL-1β (41). Thus, mitochondrial dysfunction may lead to changes in the mitochondrial membrane potential, release of mitochondrial ROS, and other mechanisms that activate the NLRP3 inflammasome and contribute to the release of IL-1β to promote gout progression. For example, a small heterodimer partner can inhibit NLRP3 activation by competing for NLRP3 binding to an apoptosis-associated speck-like protein containing CARD (ASC). Deletion of a small heterodimer partner caused an excessive NLRP3 response in a mouse model of gout *via* a mechanism involving severe damage to and accumulation of mitochondria and the sustained action of NLRP3 and ASC (42). Similarly, Raf kinase inhibitor protein negatively regulates inflammasome activation by competing for NLRP1, NLRP3, and NLPC4 inflammasome binding to ASC. The depletion of Raf kinase inhibitor protein also exacerbates gouty arthritis (43). Heat shock protein 60 (HSP60) expression is upregulated in the peripheral blood mononuclear cells and serum of patients with acute gout. MSU crystals can also induce macrophage HSP60 expression, thereby promoting the collapse of mitochondrial membrane potential and mitochondrial ROS production, leading to mitochondrial dysfunction and activation of the NLRP3 inflammasome *via* a mechanism involving the TLR4/MyD88/NF-κB signaling pathway. Knockdown or overexpression of HSP60 affects TLR4 and MyD88 expression, degradation of Ikβα, and the nuclear localization of NF-κB (44).

### Therapeutic Targets and Molecules Associated With Pyroptosis Improve Gout

Pyroptosis plays a vital role in the progression of gout and has thus been targeted because of its clinical treatment potential. Organisms have several mechanisms that negatively regulate pyroptosis. For example, an MSU-induced increase in macrophage CD44 increases caspase1 activity and IL-1β production by decreasing phosphatase 2A activity (45). Proteoglycan-4, a glycoprotein produced by synovial fibroblasts, inhibits MSU-induced inflammatory responses by interacting with CD44 receptors on macrophages, thereby reducing NF-κB nuclear translocation, NLRP3 expression, caspase1 activation, and mature IL-1β production (46). The mechanism underlying the negative regulation of pyroptosis appears insufficient to suppress the inflammatory response. Therefore, drugs that inhibit pyroptosis, either directly or indirectly, should be developed to treat gout (**Table 1**).

**Table 1:** Multiple potential compounds targeting cell death for the treatment of gout.

Name	Target	Function	Ref.
Budlein A	NLRP3	Reduces neutrophil recruitment and phagocytosis of MSU inhibits the NF-κB signaling pathway to reduce TNFα expression, and inhibits NLRP3 assembly to reduce IL-1β release.	(47)
OLT1177	NLRP3	Inhibits NLRP3 and remarkably reduces MSU-induced joint swelling and synovial IL-1β expression in mouse synovial tissue.	(48)
Arhalofenate	NLRP3	Inhibits NLRP3 and blocks URAT1.	(49)
Caffeic acid phenethyl ester	NLRP3	Directly blocks ASC binding to NLRP3, inhibiting MSU-induced NLRP3 inflammasome assembly.	(50)
Tetrahydropalmatine	NLRP3	Inhibits NLRP3 activation by increasing the antioxidant mechanism.	(51)
AI-44	NLRP3	Suppresses activation of the NLRP3 inflammasome by inhibiting the release of cathepsin B.	(52)
*Actinidia arguta extract*	NLRP3	Inhibits NLRP3 activation in experimental models of gout by regulating ASC oligomerization.	(53)
*Cinnamomum cassia extract*	NLRP3	Inhibits NLRP3 activation in experimental models of gout by regulating ASC oligomerization.	(54)
Guizhi-Shaoyao-Zhimu decoction	NLRP3	Attenuates the action of ASC with pro-caspase1 and inhibits the NF-κB signaling pathway to suppress MSU-induced NLRP3 inflammasome activation and inflammation.	(55)
Cucurbitacin B	NLRP3	Inhibits MSU-induced activation of macrophage glycolytic key enzymes and NLRP3 inflammasome and suppresses IL-1β secretion.	(56)
Resveratrol	NLRP3	Inhibits NLRP3 activation by inhibiting acetylated α-tubulin-mediated mitochondrial spatial alignment and contact with the endoplasmic reticulum induced by mitochondrial damage in macrophages, leading to insufficient NLRP3 assembly in the mitochondrial ASC and endoplasmic reticulum.	(57)
Chaetocin	NLRP3	Inhibits MSU-induced NLRP3 inflammasome activation and inflammation by suppressing the expression of HIF-1α and hexokinase 2.	(58)
Andrographolide	NLRP3	Attenuates ROS-mediated NLRP3 inflammasome assembly and IL-1β release *via* the HO-1 pathway.	(59)
Loganin	NLRP3	Inhibits MSU-induced mitochondrial damage and NLRP3 activation in macrophages by increasing mitochondrial membrane potential and reducing mitochondrial ROS.	(60)
Quercetin	NLRP3	Reduces mechanical hyperalgesia generated by MSU, leukocyte recruitment, TNFα and IL-1β generation, superoxide anion production, inflammasome activation, antioxidant depletion, NF-κB activation, and mRNA expression inflammasome components.	(61)
Procyanidin B2	NLRP3	Inhibits cathepsin B release from macrophages, NLRP3 activation, and IL-1β release and downregulates prostaglandin E2 expression.	(62)
14,2-(2-chlorobenzyl)-N-(4-sulfamoylphenethyl)acrylamide	NLRP3	Inhibits NLRP3 ATPase activity from inhibiting NLRP3 activation.	(63)
*Mollugo pentaphylla extract*	NLRP3	Inhibited TNFα, IL-1β release, and NLRP3 inflammasome activation to attenuate inflammatory paw swelling and pain in MSU-induced mice.	(64)
Paeonol	NLRP3	Reduces the synthesis of IL-1β *via* blocking the activation of the NLRP3 inflammasome, NF-κB signaling pathway, and MAPK signaling pathway.	(65)
S14G-humanin	NLRP3	Inhibits MSU-stimulated macrophage mitochondrial ROS production, malondialdehyde levels, NLRP3 inflammasome activation, and sirtuin type-1 levels.	(66)
Cichoric acid	NLRP3	Inhibits the degradation of IκBα and NF-κB signaling pathway, NLRP3 inflammasome activation, and its downstream expression levels of inflammatory genes *IL- 1β, TNF-α, COX-2*, and *PGE2* to suppress MSU-induced inflammatory responses in macrophages.	(67)
Jia-Wei-Si-Miao-Wan	NLRP3	Inhibits NLRP3 inflammatory vesicles and TLR/NF-κB signaling pathways.	(68)
MCC950	NLRP3	Turns the active conformation of NLRP3 to the inactive state.	(69)
Eucalyptol	NLRP3	Reduces ROS production and increases antioxidant enzyme activity.	(70)
Procyanidins	NLRP3	Reduce ROS levels and inhibit MSU-induced NLRP3 inflammasome activation in the mouse macrophage line RAW264.7 to improve gout pain.	(71)
Epigallocatechin gallate	NLRP3	Reduces ROS levels.	(72)
Catechin	NLRP3	Reduces ROS levels.	(73)
Baeckein E	NLRP3	Blocks MAPK/NF-κB signaling pathway and mitochondrial damage-induced oxidative stress.	(74, 75)
Curcumin	NLRP3	Blocks MAPK/NF-κB signaling pathway and mitochondrial damage-induced oxidative stress.	(74, 75)
Ru(bpy)_2_(NO)SO_3_	NLRP3	Ameliorates gouty joint inflammation and pain by inhibiting MSU-induced joint oxidative stress and pro-inflammatory cytokine levels, NF-κB activation, and IL-1β expression *via* the cGMP/PKG/ATP-sensitive K^+^ channel signaling pathway	(76)
Trans-chalcone	NLRP3	Inhibits oxidative stress, pro-inflammatory cytokines, NF-κB activation, and NLRP3 inflammasome activation.	(77)
Disulfiram	NLRP3	Inhibits lysosomal cathepsin B release and mitochondrial ROS production.	(78)
Erianin	NLRP3	Inhibits NLRP3 ATPase activity.	(79)
Tranilast	NLRP3	Inhibits NLRP3 assembly by blocking NLRP3 oligomerization through direct binding to the NACHT structural domain and shows *ex vivo* activity toward monocytes from gout patients.	(80)
β-carotene	NLRP3	Directly inhibits MSU-induced NLRP3 activation by binding to PYD.	(81)
Chrysin	NLRP3	Directly inhibits MSU-induced NLRP3 activation by binding to the PYD of NLRP3 and regulates the expression of uric acid transporter proteins.	(82)
Sulforaphane	NLRP3	Directly inhibits the activation of the NLRP3 inflammasome.	(83)
Coptisine	NLRP3	Directly inhibits NLRP3 inflammasome assembly by affecting binding between pro-caspase1 and ASC and inactivating the NF-κB pathway, thereby preventing LPS-induced IL-1β production in macrophages and MSU-induced foot tissue swelling in mice	(84)
Artemisinin	NLRP3	Inhibits the expression of NEK7 and affects the concentration of K^+^.	(85)
3β,23-dihydroxy-12-ene-28-ursolic acid	NLRP3	Inhibits NLRP3 inflammasome through PI3K/Akt/mTOR-dependent autophagy.	(86)
Rhein	Caspase1	Inhibits MSU-induced caspase1 protease activity in macrophages and suppresses IL-1β release.	(87)
Anakinra	IL-1	Can directly inhibit IL-1 and reduce NETosis.	(88)
A-769662	AMPK	Activates phosphorylation of AMPK to inhibit NLRP3 activation, caspase1 activity, and IL-1β level.	(89)
Arhalofenate	AMPK, NLRP3	Inhibits NLRP3 inflammasome through AMPK signaling and oxidative stress.	(90)
Tanshinones	AMPK, NLRP3	Reduce mitochondrial ROS, restore mitochondrial function, promote autophagy and AMPK signaling pathways to protect mitochondria, and inhibit NLRP3 inflammasome formation.	(91)
MRS2578	P2Y6	Inhibits NET formation by regulating P2Y6/store-operated calcium entry/IL-8 axis.	(92)
AS605240	PI3K-γ	Inhibits PI3K activity, increases neutrophil apoptosis, inhibits NF-κB activation, and reduces IL-1β levels.	(93)
GSK045	PI3K-δ	Inhibits PI3K activity, increases neutrophil apoptosis, inhibits NF-κB activation, and reduces IL-1β levels.	(93)
CL27c	PI3Ks	Inhibits PI3K activity, increases neutrophil apoptosis, inhibits NF-kB activation, and reduces IL-1β levels.	(93)
AT-01-KG	FRP2/ALX	Inhibits inflammation by inducing neutrophil apoptosis and cytotoxicity.	(94)
(*E*)-1-(6-methoxybenzo[d]oxazol-2-yl)-2-(4-methoxyphenyl)ethanone oxime	NLRP3, xanthine oxidase	Can inhibit xanthine oxidase activity, TLR4 expression, and NLRP3 activation.	(95)
Hydrogen sulfide	Caspase1, NLRP3, xanthine oxidase	Inhibits xanthine oxidase/caspase1 activity, mitochondrial ROS production, and ASC oligomerization.	(96)
*Chrysanthemum indicum extract*	Inflammasomes	Inhibits LPS-induced inflammasome macrophage activation, reduces IL-1β secretion, and attenuates neutrophil aggregation by regulating ASC phosphorylation.	(97)
Sulforaphane	Inflammasomes	Can inhibit the activation of multiple inflammasomes and reduce caspase1 activity through an Nrf2-independent mechanism.	(98)
Methylene blue	Inflammasome, caspase1	Inhibits the activation of multiple inflammasomes and reduces caspase1 activity through the NF-κB signaling pathway.	(99)

Several existing natural ingredients and artificial pharmacological inhibitors improve gout by directly inhibiting the assembly or activation of the NLRP3 inflammasome. For example, erianin showed *ex vivo* activity in synoviocytes and monocytes of patients with gout. Erianin inhibits the assembly of NLRP3 by directly interacting with it and is associated with the Walker A motif in the NACHT structural domain, inhibiting NLRP3 ATPase activity (79). Gavage of sulforaphane in a mouse model of acute gout revealed that sulforaphane reduced MSU-induced tissue swelling and inflammatory cell infiltration in the mouse foot by directly inhibiting NLRP3 inflammasome activation independent of ROS (83). The mRNA expression of NIMA-related kinase 7 (*NEK7*) and *NLRP3* is upregulated in gout patients (85). NEK7 is an essential component of NLRP3 inflammasome (100). Artemisinin inhibits LPS- and MSU-induced NEK7 and NLRP3 expression levels in macrophages and attenuates K^+^ efflux from macrophages to reduce foot and ankle swelling in mice with arthritis (85).

In addition, some mechanisms indirectly inhibit MSU activation of the NLRP3 inflammasome and thus prevent its activation by restoring mitochondrial function and reducing ROS production. Many antioxidants derived from natural sources are responsible for this effect. Eucalyptol inhibited inflammatory cell infiltration, upregulation of TRPV1 expression, activation of the NLRP3 inflammasome, and production of pro-inflammatory cytokines induced by MSU injection in mouse ankle joints to suppress gout and joint inflammation primarily through antioxidant mechanisms. The underlying mechanisms included reducing ROS production and increasing antioxidant enzyme activity (70). Epigallocatechin gallate and catechin are tea-like bioactive polyphenols. Epigallocatechin gallate can inhibit MSU-induced neutrophil infiltration, NLRP3 expression, and pro-inflammatory factor secretion to ameliorate inflammation (101). Epigallocatechin gallate inhibits *de novo* mitochondrial DNA synthesis and ROS production in mouse macrophages and the NLRP3 inflammasome and prevents gouty inflammation (72). Catechin inhibits NLRP3 inflammasome activation by upregulating the mitochondrial survival protein BCL2 level in monocytes, restoring MSU-induced impairment of the mitochondrial transmembrane potential, and reducing intracellular ROS and Ca^2+^ levels (73). In addition to natural antioxidants, other substances have similar effects. For example, soluble decoy receptor 3 (DcR3) promotes the differentiation of anti-inflammatory M2 macrophages and inhibits NLRP3 activation by reducing MSU-induced mitochondrial dysfunction and lysosomal rupture to decrease ROS production and cathepsin levels and to improve gout (102). Disulfiram inhibits NLRP3 activation by inhibiting the release of lysosomal histone B into the cytoplasmic lysate and inhibiting mitochondrial ROS production and has significant efficacy against MSU-induced gout inflammation (78). Interestingly, another study tested the degree of NLRP3 activation in MSU-stimulated mouse macrophages at different temperatures (25, 33, 37, 39, and 42°C) and found that 37°C was the optimal temperature. Greater NLRP3 activation was observed at lower temperatures, indicating that low temperatures contribute to the MSU-induced activation of the NLRP3 inflammasome. Therefore, thermotherapy may be an effective form of physiotherapy for gout (103).

IL-1 inhibitors, such as anakinra, have also been shown to ameliorate gout. A pilot, open-label study (trial registration number ISRCTN10862635) evaluated the efficacy of anakinra in 10 patients with gout who were intolerant or had failed other conventional therapies and found that all patients responded well to anakinra without demonstrating any adverse effects (88). Another clinical trial on the efficacy and safety of anakinra in 40 patients with gout revealed that most responded well to anakinra, suggesting that it can be used as an alternative to other therapies or for short-term use. However, long-term administration is associated with complications, such as infection (104). MSU-activated NLRP3 inflammasomes induce IL-1β and IL-1α release. NLRP3 inflammasomes are required to activate calpain and the intracellular cleavage of IL-1α, thereby aiding IL-1α release (105, 106). Given the efficacy of IL-1 receptor antagonists in gout, neutralizing antibodies against IL-1α may also be beneficial. Notably, targeted inhibition of the pyroptosis effector protein gasdermin D may not be as necessary for MSU-induced IL-1β release as gasdermin D mediated membrane destruction. MSU may also disrupt cells through other mechanisms, leading to IL-1β release (107). Therefore, studies on the link between pyroptosis and gout should focus on upstream mechanisms, such as the NLRP3 inflammasome.

### Effect of Crosstalk Between Autophagy and Pyroptosis on Gout

**Figure 2:**
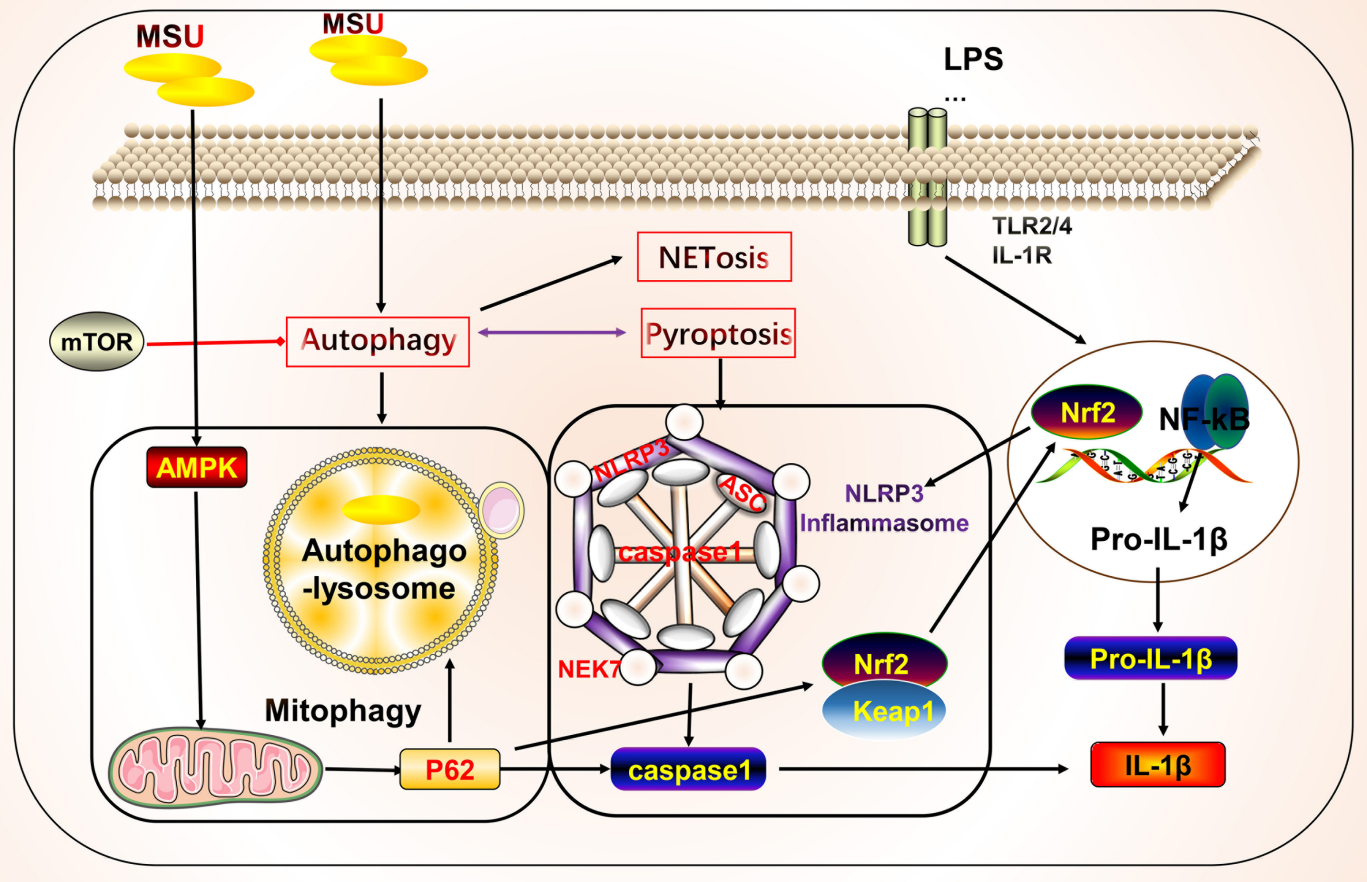
Crosstalk between autophagy and pyroptosis on gout. Autophagy is primarily associated with pyroptosis in gout. Autophagy induced by monosodium urate (MSU) leads to the formation of autophagolysosomes that engulf MSU to isolate further inflammatory responses. AMPK is involved in autophagy regulation by regulating mitochondrial function. The autophagy protein P62 plays a dual role, participating in mitochondrial autophagy with anti-inflammatory effects and in crosstalk with pyroptosis to promote inflammation. Autophagy also cross-talks with other cell death pathways, such as NETosis.

Autophagy is a conserved cellular pathway that controls the degradation of proteins and organelles and plays a vital role in maintaining homeostasis and disease progression (108). Its molecular mechanisms have been extensively reviewed, primarily phosphorylation involving the mammalian target of rapamycin (mTOR), autophagy initiation recognition, formation of autophagy-induced complexes, and formation of final degradation products of autophagic lysosomes (11). Autophagy contributes to the pathogenesis of gout through crosstalk with pyroptosis (**Figure 2**). Osteoblasts phagocytose MSU, causing upregulation of NLRP3 activation to promote autophagy and autophagosome formation by reducing mTOR levels and promoting LC3-II cleavage, but not IL-1β release. This phagocytosis may prevent the harmful effects of MSU by maintaining it inside the autophagosome (109). In addition, *Cyclocarya paliurus* ameliorates hyperuricemia and gout *via* a mechanism that may involve 3β,23-dihydroxy-12-ene-28-ursolic acid, a potent substance derived from *C. paliurus* that inhibits NLRP3 inflammasome formation *via* PI3K/Akt/mTOR-dependent autophagy (86). Similarly, resveratrol improves gout inflammation by upregulating sirtuin1 expression, inhibiting the mRNA expression of NLRP3 and NF-κB, and promoting autophagy (110).

We focused on the roles of AMP-activated protein kinase (AMPK) and P62 in the crosstalk between autophagy and pyroptosis. Protein kinase AMP-activated non-catalytic subunit gamma 2 (*PRKAG2)* (cg09817217 and cg07012178), encoding the γ2 chain of AMPK, showed a gene body hypomethylation pattern in peripheral blood mononuclear cells of gouty arthritis patients (111). MSU inhibits AMPKα phosphorylation in macrophages *in vitro*. The AMPK activator, A-769662, inhibits the inflammatory response to MSU by promoting AMPK-dependent polarization of the M2 anti-inflammatory macrophage phenotype and AMPK kinase phosphorylation, inhibiting NLRP3 expression, and suppressing caspase1 and IL-1β activation. *In vitro* intervention with 10 nM colchicine resulted in effects similar to those of A-769662 (89). Colchicine can also inhibit NLRP3 inflammasome signaling by irreversibly inhibiting tubulin polymerization and microtubules (112). Arhalofenate enhances AMPK activity in macrophages to regulate mitochondrial function and oxidative stress-related AMPK downstream gene expression, inhibits MSU-induced NLRP3 inflammasome activation and IL-1β release, and promotes autophagic flux to suppress inflammation (90). Tanshinones attenuate LPS-induced mitochondrial ROS production, restore mitochondrial function, promote autophagy and AMPK signaling pathways to protect the mitochondria, and inhibit NLRP3 inflammasome formation in macrophages (91). P62 is a selective autophagy receptor that plays a dual role in gout. NF-κB induces the expression of pro-IL-1β and NLRP3 to initiate the assembly of NLRP3 inflammasomes. NF-κB also induces a specific mechanism for the delayed accumulation of P62/SQSTM1 during inflammation. When NLRP3 is activated and the mitochondria are damaged, the damaged mitochondria undergo parkin-dependent ubiquitin-binding and are thus recognized by P62, which then undergoes cell-selective autophagy and mitochondrial autophagy, thereby clearing damaged mitochondria to prevent the release of mitochondrial ROS and other substances to suppress inflammation (113). By contrast, MSU stimulates autophagosome formation, leading to impaired proteasomal degradation and P62 accumulation. Accumulation of P62 leads to IL-1β and caspase1 expression through activation of extracellular signal-regulated kinase and JNK signaling. IL-1β, in turn, induces the P62 protein and creates a vicious cycle; additionally, the silencing of Atg16L1, a protein essential for autophagosome formation, leads to P62 accumulation and enhances inflammation (114). In addition, P62 promotes its nuclear translocation by binding to Kelch-like ECH-associated protein 1 and releasing nuclear factor erythroid 2-related factor 2, which induces the transcription of heme oxygenase-1 and superoxide dismutase, thereby activating the NLRP3 inflammasome (115, 116). Thus, when mitochondrial autophagy functions effectively, P62 can inhibit NLRP3 activation by suppressing inflammation. However, when mitochondrial autophagy is inadequate or impaired, P62 is produced in large amounts and promotes inflammation.

## NETosis

**Figure 3:**
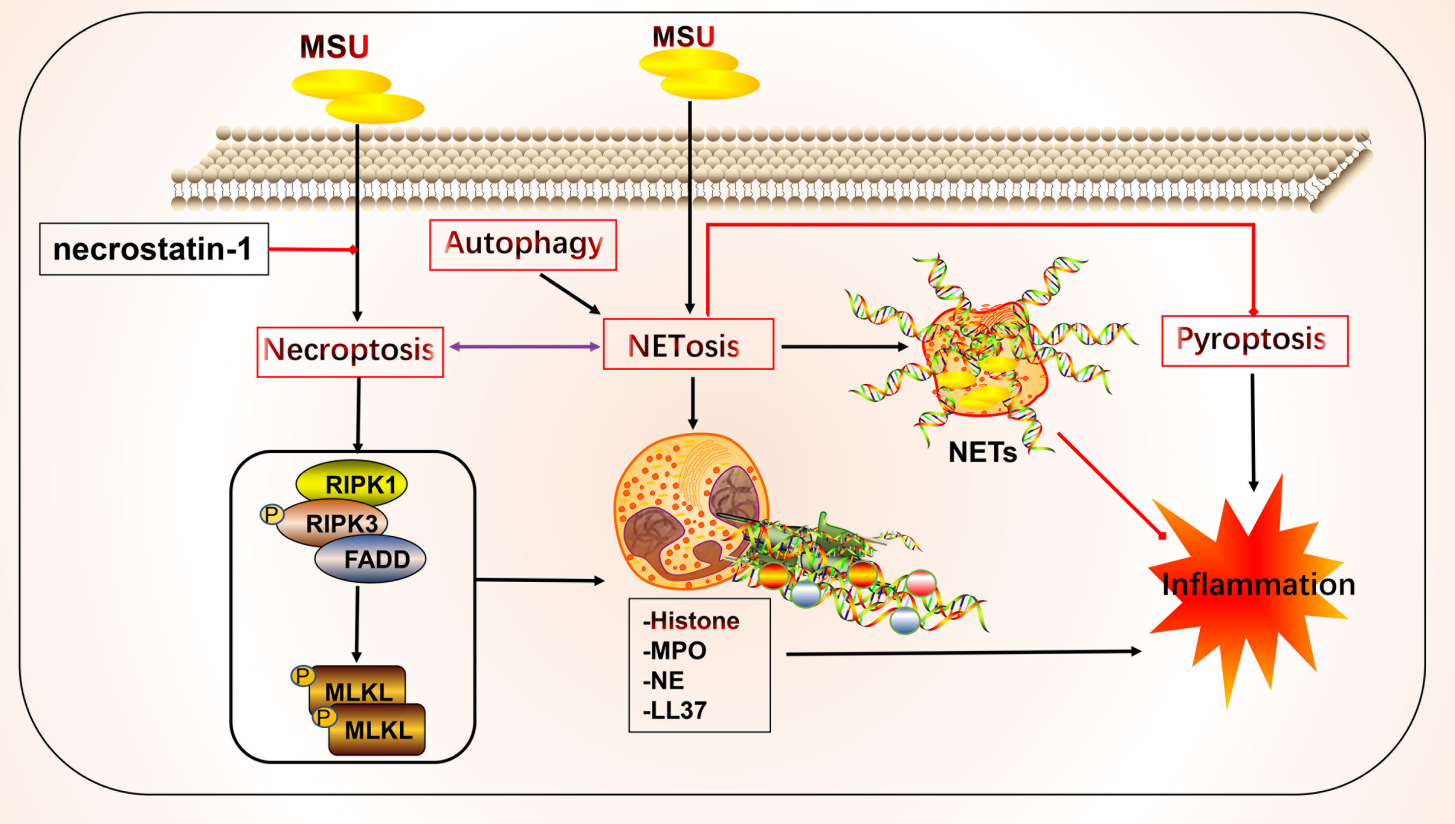
Crosstalk of NETosis with other forms of cell death and implications for gout. Monosodium urate (MSU) -stimulated NETs require key molecules involved in necroptosis. Neutrophils can promote cytokine degradation by forming NETs, phagocytosing MSU, and physically isolating them to suppress inflammation. Histones released during NETosis may have cytotoxic and immunostimulatory effects on cells. In addition, crosstalk with other forms of cell death, including pyroptosis and autophagy, may occur.

Neutrophils can participate in gout inflammation and gout stones by inducing the formation of neutrophil extracellular traps (NETs), which release a range of mediators including histones, ROS, and a variety of proteases (117, 118). NETs are extracellular meshworks containing chromatin and granule proteins and were initially considered a means of targeting microorganisms. These features include disassembly of nuclear and granule membranes and hypercitrullination of histones by peptidylarginine deiminase 4, which mediates chromatin decondensation, membrane rupture, and concomitant ROS production by NADPH oxidase (119). ROS trigger the release of neutrophil elastase into the cytoplasm and activate neutrophil elastase enzymatic activity in a myeloperoxidase-dependent manner, contributing to its degradation of F-actin and translocation to the nucleus for continued degradation of core histones, such as H1 and H4, subsequently promoting chromatin depolymerization and NETosis (118, 120–122) (**Figure 3**).

NETs and NETosis may play both anti-inflammatory and pro-inflammatory roles in gout. Histones released during NETosis may be important in the progression of gout and its related clinical symptoms; however, histones exert cytotoxic and immunostimulatory effects on glomerular cells (123), have cytotoxic effects, and increase lethality in endothelial cells and mouse models of sepsis *in vitro* (124). By contrast, MSU can trigger NET formation and NETosis. In standard synovial fluid from MSU-induced arthritis, non-refractive fibrous material, cells, and crystals were observed that primarily consisted of fibrin and collagen fibers that formed NETs (125). MSU and low-grade inflammation are often detected in the synovial fluid of joints of patients with asymptomatic gout, possibly in a state of clinical sub-inflammation (126, 127). Although MSU is thought to be an essential driver of inflammation, in practice it may not lead to a robust inflammatory response, which is consistent with the disease profile of clinically asymptomatic patients with gout. The robust inflammatory response also requires stimulation by other factors that lead to intense inflammation, such as the NLRP3 inflammasome (128–131). In this process, NETs may act as a physical barrier by phagocytosing MSU, thus forming a large reticulation of NETs to prevent the spread of harmful substances and influence disease progression (125). Studies have shown that neutrophils that phagocytose MSU form NETs by extruding DNA, packing crystals, and degrading cytokines to suppress excessive inflammatory responses (132). Additionally, NETs limit the inflammatory response by degrading cytokines and chemokines and inhibiting neutrophil recruitment and activation; however, the onset of NET formation leads to inflammation (133, 134).

NETs and NETosis may be linked to necroptosis (135). The critical molecules involved in necroptosis include RIPK1, RIPK3, and MLKL. RIPK1 and RIPK3 form a complex and activate MLKL, which leads to membrane pore formation, membrane leakage, and cell lysis, resulting in the release of cellular components. The detailed molecular mechanisms underlying these effects have been previously reviewed (11, 136, 137). Mulay et al. found that crystals of calcium oxalate, MSU, calcium pyrophosphate dihydrate, and cystine induce cysteinase-independent cell death in renal tubular epithelial cells, which is blocked by the RIPK1 inhibitor necrostatin-1, suggesting that crystal-induced cell death is associated with necroptosis (138). Garcia-Gonzalez et al. evaluated NETs in the synovial fluid of patients during a gout attack. Neutrophils in the synovial fluid release NETs in response to MSU, depending on the number of crystals, but not on cell density, which is accompanied by the activation of MLKL, a key molecule of necroptosis (125). Similarly, MSU-induced NETs were accompanied by RIPK3 expression and MLKL phosphorylation, and the inhibition of RIPK1, RIPK3, and MLKL inhibited NET formation in human and mouse neutrophils (139). The bridge linking the two forms of death may be ROS. Preferential activation of the three critical molecules of necroptosis leads to membrane rupture in neutrophils and ROS production, stimulating NET formation and NETosis (139). Thus, in addition to the association between necroptosis and NETs, pyroptosis of macrophages and NETs in neutrophils are interlinked. The latter may be a compensatory measure to suppress inflammation *via* NET formation (140). Sil et al. found that macrophage-derived IL-1β enhances the formation of MSU-stimulated NETs. Anakinra reduced NETosis in macrophages. In addition, caspase11-deficient experimental mice with gout and their source macrophages responded to MSU stimulation by significantly reducing the production of cytokines, such as IL-1β, TNFα, and IL-6. Caspase11-deficient neutrophils also failed to produce NETs, the mechanism of which may involve the effect of caspase11 on the regulation of macrophage pyroptosis in gout and NET formation (141). The P2Y6 receptor in endothelial cells *in vitro* activates LPS-induced transendothelial migration of neutrophils *via* Rho kinase (142). The P2Y6 receptor antagonist MRS2578 inhibits MSU-induced NETosis *via* a mechanism that may involve the P2Y6/store-operated calcium entry/IL-8 axis (92). In addition to the aforementioned link between NETosis, necroptosis, and pyroptosis, mutual crosstalk with autophagy may occur. Nuclear factor, interleukin 3 regulated (NFIL3), which is highly expressed in neutrophils of patients with gout, promotes its transcription by binding to the REDD1 promoter, which enhances autophagy and NET formation by inhibiting the mTOR pathway, to promote inflammatory responses (143). Inhibition of autophagy to prevent intracellular chromatin depletion leads to apoptosis, because intracellular chromatin depletion is necessary for NET formation and NETosis (144). Neutrophils from patients with gout form NETs in an autophagy-dependent manner, and inhibition of phagolysosome and PI3K signaling pathways prevents MSU-induced NETs and NETosis (145).

## Apoptosis

**Figure 4:**
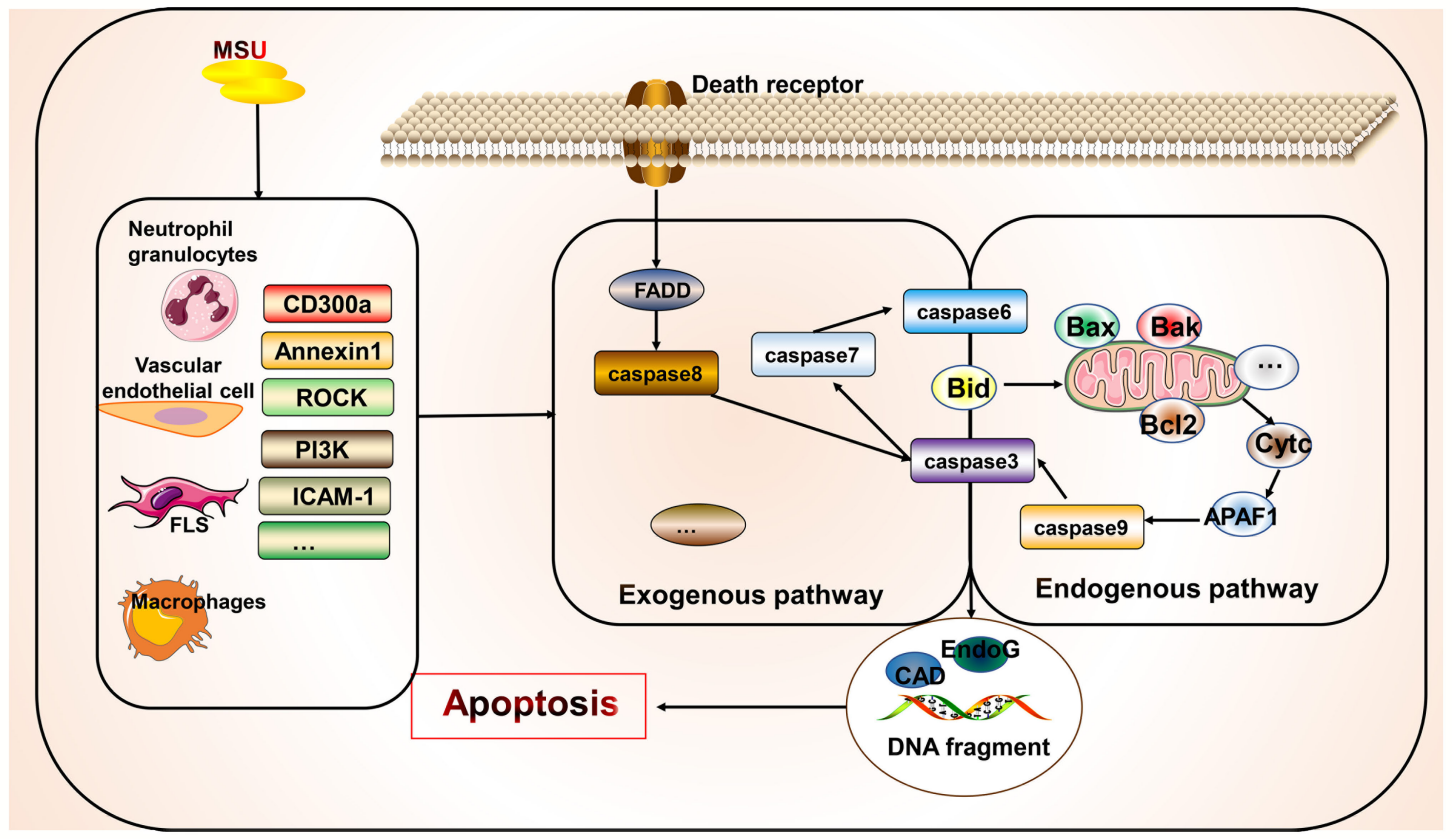
Association of apoptosis with gout. Apoptosis occurs primarily *via* endogenous and exogenous pathways. Apoptosis exists in various cells in gout but has not been widely examined. However, the managing gout involves inhibiting the apoptosis of beneficial cells and promoting the apoptosis of harmful cells. Some apoptosis-related molecular targets such as CD300a have been identified and require further investigation.

Apoptosis is a controlled form of cell death that primarily involves the exogenous death receptor and endogenous mitochondrial pathways. Its key molecules include multiple death receptors, caspase enzymes, and mitochondrial proteins that mediate apoptosis (11). The morphological features of apoptosis include cytoplasmic concentration, breakage of DNA, and formation of apoptotic vesicles. This process generally does not result in the release of cellular contents. However, many apoptotic cells lead to inefficient clearance, causing an inflammatory response (11). However, few studies have directly examined the relationship between gout and apoptosis (**Figure 4**). Studies have shown that new macrophages are constantly recruited within the gout tophi and that these macrophages produce TNF-α and matrix metalloproteinase-2/9 to induce degradation of the stroma. However, apoptosis occurs in CD68^+^ macrophages, which limits inflammation (146). MSU stimulation can affect apoptotic processes in various cells and gout through different mechanisms, with most studies focusing on apoptosis in neutrophils and endothelial cells. MSU stimulates the release of ROS and reactive nitrogen species from fibroblast-like synoviocytes, leading to endoplasmic reticulum oxidative stress and possibly mitochondrial dysfunction, thereby promoting fibroblast-like synoviocyte apoptosis (147). The adhesion between neutrophils and endothelial cells is key to the development of acute gout (148). MSU is also gradually deposited on the vessel wall to induce a vascular inflammatory response and damage the patient’s vascular endothelial cells, thereby causing gout complications. During inflammation regression, neutrophils migrating to the inflammatory site first undergo apoptosis and are phagocytosed by phagocytic cells (149). Some apoptosis-related mechanisms in the body improve inflammation in order to cope with the inflammatory response in gout. Promoting the apoptosis of neutrophils and inhibiting the apoptosis of endothelial cells are beneficial for suppressing the inflammatory response to improve gout. CD300a is an inhibitory receptor whose expression increased in neutrophils from the knees of mice after MSU stimulation, which promotes apoptosis of neutrophils by increasing the cleavage of caspase8 of the exogenous death receptor pathway (150). Similarly, annexin A1, a glucocorticoid regulatory protein, reduced neutrophil infiltration and IL-1β release by inducing apoptosis in neutrophils in a MSU-induced gouty arthritis mouse model (151). Although multiple anti-inflammatory mechanisms exist, their actions may be insufficient to inhibit inflammation. Therefore, promoting endogenous anti-inflammatory substances or inhibiting the production of pro-inflammatory molecules—and thus promoting inflammatory cell apoptosis or inhibiting beneficial cell apoptosis through artificial agonists or inhibitors—is a potential future therapeutic direction. For example, inhibition of Rho-associated kinase, a serine/threonine kinase, attenuates neutrophil accumulation, IL-1β levels, and hypernociception in experimental models of gout through mechanisms involving reduced MYPT phosphorylation, decreased Iκβα activity, and increased caspase-dependent apoptosis of neutrophils (152). Similarly, PI3K inhibitors AS605240, GSK045, and CL27c significantly reduced MSU-induced neutrophil infiltration by increasing apoptosis and reducing myeloperoxidase activity, NF-κB activation, and IL-1β levels, thus improving inflammation (93). Simiaowan reduced neutrophil infiltration, inhibited endothelial cell apoptosis in rats with MSU-induced gout, and improved the inflammatory response by attenuating the expression of ICAM-1 (148). AT-01-KG, an artificial agonist of FRP2/ALX, inhibits inflammation by inducing neutrophil apoptosis and cytarabine in a mouse model of gouty arthritis (94). Natural traditional medicines have also been shown to inhibit apoptosis of chondrocytes in gouty arthritis, as summarized in a previous review (153). *Anemonoides nemorosa* and *Tecomella undulata* are used in traditional medicines to treat gout. Studies showed that Aqueous extracts of *A. nemorosa* can induce cell cycle arrest and apoptosis by regulating mitochondrial function (154). *Tecomella undulata* induces apoptosis in tumor cells by promoting Fas, Fas-associated death domain, caspase3/7/8, and DNA fragmentation (155). In addition, some clinical medications for gout are effective in modulating apoptosis. Uromodulin gene mutations cause premature hyperuricemia, gout, and kidney damage (156). Uromodulin gene mutations affect the processing of uromodulin/tammhorsfall glycoprotein (156). Colchicine promotes the secretion of tammhorsfall glycoprotein from the endoplasmic reticulum, which significantly reduces apoptosis, whereas intracellular endoplasmic reticulum accumulation of tammhorsfall glycoprotein promotes apoptosis (156). Allopurinol reduces apoptosis and increases osteoblast viability in an animal model of hyperuricemia and reduces the risk of vascular calcification by decreasing *Wnt3a*, *Runx2*, *Sp7, Bglap*, *Col1a1*, *SM22a*, and *Acta2* expression in vascular smooth muscle cells (157).

## Conclusion

Cell survival and death are fundamental processes. The molecular mechanisms and morphological characteristics of various types of cell death have been revealed to be closely related to multiple diseases. First, we explored the role of pyroptosis in gout, in which the assembly of NLRP3, a key molecule in pyroptosis, is considered a critical step in the progression from hyperuricemia to gout. In response to MSU stimulation, NLRP3 inflammasomes undergo assembly and release IL-1β, which promotes inflammation. Second, we discussed the crosstalk between NETosis and other forms of cell death and its relationship with gout. NETosis appears to require the assistance of crucial molecules involved in necroptosis for activation. The occurrence of NETs and NETosis in neutrophils appears to be a compensatory mechanism that suppresses inflammation. Finally, we described the roles of these two forms of cell death in gout, autophagy, and apoptosis, which have not been widely evaluated and require further investigation. Studies should focus on the impact of crosstalk between different forms of cell death on gout pathogenesis. In addition, using artificial agonists and inhibitors to target cell death or biological mediators that regulate cell death is a promising therapeutic approach. Whether the combined targeting of multiple pathways and molecules is beneficial for intercellular crosstalk should be examined. Forms of cell death other than those covered in this study may exist and be relevant to gout pathogenesis; however, current research seems to be limited. Thus, unexplored forms of cell death will remain a potential direction of future research. Although translating preclinical results into clinical practice remains challenging, there is great potential to target the precise mechanisms of cell death in gout.

## Author Contributions

JZ is responsible for the collection, collation and writing of the original manuscript. KW, PJ, CC, LXX, LSX and YS is responsible for the collection of the original manuscript. SG, YX, and DH are responsible for the revision and review of the manuscript. All authors reviewed and accepted with the final version.

## Funding

This work was funded by the National Natural Science Funds of China (82074234 and 82071756), Shanghai Chinese Medicine Development Office, National Administration of Traditional Chinese Medicine, Regional Chinese Medicine (Specialist) Diagnosis and Treatment Center Construction Project-Rheumatology, State Administration of Traditional Chinese Medicine, National TCM Evidence-Based Medicine Research and Construction Project, Basic TCM Evidence-Based Capacity Development Program, Shanghai Municipal Health Commission, and East China Region-based Chinese and Western Medicine Joint Disease Specialist Alliance.

## Conflict of Interest

The authors declare that the research was conducted in the absence of any commercial or financial relationships that could be construed as a potential conflict of interest.

## Publisher’s Note

All claims expressed in this article are solely those of the authors and do not necessarily represent those of their affiliated organizations, or those of the publisher, the editors and the reviewers. Any product that may be evaluated in this article, or claim that may be made by its manufacturer, is not guaranteed or endorsed by the publisher.

## Abbreviations

NLRP3, the NACHT, LRR, and PYD domains-containing protein 3; MSU, monosodium urate; TNF, tumor necrosis factor; IL, interleukin; ASC, apoptosis-associated speck-like protein containing a C-terminal caspase activation and recruitment domain; ROS, reactive oxygen species; TLR, Toll-like receptor; MyD, myeloid differentiation primary response; NF-κB, nuclear factor kappa B; RIPK1, receptor interacting serine/threonine kinase 1; RIPK3, receptor interacting serine/threonine kinase 3; MLKL, mixed lineage kinase domain-like protein; LPS, lipopolysaccharide; TRPV4, transient receptor potential vanilloid 4; PI3K, phosphatidylinositol-3-kinase; C5aR2, C5a receptor 2; C5aR1, C5a receptor 1; HSP60, heat shock protein 60; NEK7, NIMA-related kinase 7; DcR3, decoy receptor 3; mTOR, the mammalian target of rapamycin; AMPK, AMP-activated protein kinase; PRKAG2, protein kinase AMP-activated non-catalytic subunit gamma 2; NETs, neutrophil extracellular traps; NFIL3, nuclear factor, interleukin 3 regulated.
